# The activation of galanin receptor 2 plays an antinociceptive effect in nucleus accumbens of rats with neuropathic pain

**DOI:** 10.1186/s12576-021-00790-5

**Published:** 2021-02-05

**Authors:** Yan Dong, Chong-Yang Li, Xiao-Min Zhang, Ya-Nan Liu, Shuang Yang, Meng-Nan Li, Shi-Lian Xu

**Affiliations:** 1 Department of Physiology, School of Basic Medicine, Kunming Medical University, Kunming, 650500 Yunnan China; 2 Department of Oncology, Affiliated Hospital, Yunnan University, Kunming, Yunnan China

**Keywords:** GalR2, Galanin, Antinociceptive effect, Neuropathic pain, Nucleus accumbens

## Abstract

Our previous research has shown that galanin plays an antinociceptive effect via binding to galanin receptors (GalRs) in nucleus accumbens (NAc). This study focused on the involvement of GalR2 in galanin-induced antinociceptive effect in NAc of neuropathic pain rats. The chronic constriction injury of sciatic nerve (CCI) was used to mimic neuropathic pain model. The hind paw withdrawal latency (HWL) to thermal stimulation and hind paw withdrawal threshold (HWT) to mechanical stimulation were measured as the indicators of pain threshold. The results showed that 14 and 28 days after CCI, the expression of GalR2 was up-regulated in bilateral NAc of rats, and intra-NAc injection of GalR2 antagonist M871 reversed galanin-induced increases in HWL and HWT of CCI rats. Furthermore, intra-NAc injection of GalR2 agonist M1145 induced increases in HWL and HWT at day 14 and day 28 after CCI, which could also be reversed by M871. Finally, we found that M1145-induced antinociceptive effect in NAc of CCI rats was stronger than that in intact rats. These results imply that the GalR2 is activated in the NAc from day 14 to day 28 after CCI and GalR2 is involved in the galanin-induced antinociceptive effect in NAc of CCI rats.

## Introduction

Neuralgia is a chronic pain which deteriorates the quality of life of patients severely. Currently, the effective treatment of Neuralgia is lacking because of its complex pathogenesis. Analgesics such as N-methyl-D-aspartic acid (NMDA) receptor antagonists, sodium channel blockers, especially opioid analgesics are used to alleviate neuropathic pain, but the severe side effects have greatly limited their universal use. Thus, the treatment of neuropathic pain has always been a major goal in the clinic.

An early study showed that intense chemical or thermal noxious stimulation-induced pain perception was modulated by an ascending nociceptive control which depended on both opioid and dopamine links in the nucleus accumbens (NAc), suggesting that the NAc might play a role in mediating the suppression of tonic or persistent pain [1]. After that, more and more studies suggested that NAc is an important limbic structure of the brain with important roles in pain modulation [2–5]. Our previous studies demonstrated that the expression of galanin was up-regulated in the NAc of rats after chronic constriction injury of sciatic nerve (CCI), and galanin played an antinociceptive effect via binding to galanin receptors (GalRs) [6].

Galanin is widely distributed in the central nervous system (CNS) and peripheral tissues with multiple biological functions**.** Galanin plays an important role in the transmission and modulation of nociceptive information in the CNS. Studies showed that exogenous galanin had an antinociceptive effect in central nucleus of the amygdala [7] and the anterior cingulate cortex (ACC) [8] in normal and neuropathic rats. The injection of galanin into ACC also induced an antinociception in rats with acute inflammation [9]. Our previous studies also demonstrated that the intra-NAc injection of galanin had an antinociceptive effect in carrageenan-induced inflammatory pain models [10] and CCI-induced neuropathic pain models [6], and which could be blocked by non-selective GalRs antagonist galantide, suggesting that the galanin-induced antinociceptive effect might be mediated by GalRs [10].

There are three cloned GalRs: GalR1–3 [11]. Our earlier research demonstrated that intra-NAc injection of GalR1-specific agonist M617 had an antinociceptive effect in CCI rats, but the analgesic effect of M617 was weaker than that of galanin, it is suggested that in addition to GalR1, other GalRs might also be involved in the analgesic effect caused by galanin [12]. In this study, we focused on the potential role of GalR2 in the galanin-induced antinociceptive effect in NAc of CCI rats. M1145 [(Arg-Gly)_2_-Asn-galanin(2–13)-Val-Leu-(Pro)_3_-(Ala-Leu)_2_-Ala-amide], is demonstrated to be a selective agonist of GalR2 [13, 14]. Removal of the N-terminal glycine residue of galanin together with a C-terminal substitution resulted in the GalR2-selective antagonist M871 [galanin(2–13)-Glu-His-(Pro)_3_-(Ala-Leu)_2_-Ala-amide] [15]. In the present study, we use the M1145 and M871 to investigate whether GalR2 activation plays an antinociceptive effect in NAc of rats with experimental sciatic neuropathy.

## Materials and methods

### Chemicals

Solutions containing (1) 0.5 nmol galanin (rat galanin, Tocris, Bristol, United Kingdom); (2) 0.05, 0.5 or 1 nmol M1145 (Tocris, Bristol, United Kingdom) were prepared in 1 μl of 0.9% sterilized saline; (3) 1 nmol M871 (Tocris, Bristol, United Kingdom) was dissolved in 1 μl of 3% acetonitrile for intra-NAc injection.

### Animal preparation and ethics statement

Male Sprague-Dawley rats (180–250 g) were obtained from the Experimental Animal Center of Kunming Medical University (Kunming, China) and housed individually on a 12-h reversed light/dark cycle, the room temperature was kept at 22 ± 1 °C. All rats had unrestricted access to water and food. The experimental protocols were tested in accordance with the approval of “the Animal Care and Use Committee at Kunming Medical University” (Approval No. KMMU2014004) and “the National Institute of Health Guide for the Care and Use of Laboratory Animals”.

### Mononeuropathic animal model

The experimental protocols of CCI for duplicating a model of mononeuropathic pain in rat were performed as previously described, which was characterized by spontaneous burning pain combined with hyperalgesia and allodynia to mechanical and/or thermal stimulation [16]. Rat was anaesthetized with intraperitoneal sodium pentobarbital (50 mg/kg). The left sciatic nerve was exposed for about 1.0 cm at the middle of thigh and then was ligated four loose knots with 4-0 chromic catgut at a 1.0 mm interval between each of them. The ligations were carefully manipulated so that the nerve was barely constricted. The skin incision was sutured with 4–0 silk sutures.

In the sham group, the same operation protocols were performed with the exception of the ligations of the sciatic nerve. In both groups, the contralateral limb (right side) remained un-operated.

### Behavioural tests

The hind paw withdrawal latency (HWL) in response to thermal stimulation and the hind paw withdrawal threshold (HWT) in response to mechanical stimulation were assessed according to previously described methods [6, 10, 12, 17].

The HWL was assessed by the hot-plate (YLS-6B, China). The temperature of the hot-plate was kept at 52 ± 0.2 °C. The unilateral hind paw of rat was put on the hot-plate gently and made sure that the entire ventral surface touched the hot-plate. The time to hind paw withdrawal was measured in seconds (s) and recorded to as the HWL to thermal stimulation.

The Randall Selitto test was used to assess the HWT to mechanical stimulation. A wedge-shaped pusher with a loading rate of 30 g/s was applied to the dorsal surface of the rat’s hind paw and the magnitude of the mechanical stimulation required to initiate the struggle response was measured.

Before the experiment, the rats were acclimated to behavioural tests for 4–5 days (d), and HWL to thermal stimulation was normally between 3 and 6 seconds (s), the HWT to mechanical stimulation was maintained between 4 and 7 grams (g).

### Procedures for intra-NAc injection

The rat was anaesthetized by an intraperitoneal injections of sodium pentobarbital (50 mg/kg) and placed on a stereotaxic instrument. A stainless-steel guide cannula with an outer diameter of 0.8 mm was inserted into the NAc (anterior to bregma: 1.7 mm; left or right of the midline: 1.6 mm; ventrally: 7.0 mm) [18] and was fixed to the skull with dental acrylic. The rats were allowed to recover from the operation for 2–3 days.

On the day of the experiment, each HWL and HWT were measured 3 times which were averaged to obtain a mean value as the baseline HWL and HWT. Each HWL or HWT test should be 5 min (min) apart from the last test to prevent discomfort or injury. Then a stainless-steel needle with an outer diameter of 0.4 mm was inserted into the guide cannula at a depth of 1–1.5 mm for intra-NAc injection. The HWL and HWT were tested 5, 10, 15, 20, 30, 45 and 60 minutes (min) after intra-NAc injection, and each HWL or HWT was recorded as a percentage change from the baseline.

At the end of the experiments, the injection sites were confirmed by histological examination. Only the data from the rats in which the needle tip was located in the NAc were used for statistical analysis.

### Western blot assay

Rats were deeply anaesthetized with 4% isoflurane and euthanized, and the bilateral NAc tissues were collected for biochemical assays. The Western blot assay procedures were performed as described previously [10, 12]. Primary antibody against GalR2 (rabbit polyclonal, 1:2000, Abcam, Cambridge, United Kingdom) was used. The expression of glyceraldehyde-3-phosphate dehydrogenase (GAPDH) (mouse monoclonal, 1:1000, Cell Signaling Technology, Boston, USA) was also determined as an internal control. The relative band density was detected by ImageJ software. Western blotting of the protein samples was repeated at least 3 times.

### Statistical analysis

The data are presented as the mean ± SEM. and analysed using GraphPad Prism 5 software. The data from the behavioural tests were assessed by two-way repeated-measures ANOVA followed by Bonferroni post-test or one-way ANOVA followed by Bonferroni's multiple comparison test or two-tailed Student's t-test. The results of Western blot were assessed by one-way ANOVA followed by Bonferroni's multiple comparison test. In two-way repeated-measures ANOVA followed by Bonferroni post-test analysis, F = between-group mean square (MS between-group) / intra-group mean square (MS intra-group), and in one-way ANOVA followed by Bonferroni's multiple comparison test, paired t-tests are used to compare difference between each two groups. *P* < 0.05 was considered statistically significant.

## Results

### Expression of GalR2 in the NAc of CCI rats

**Fig. 1:**
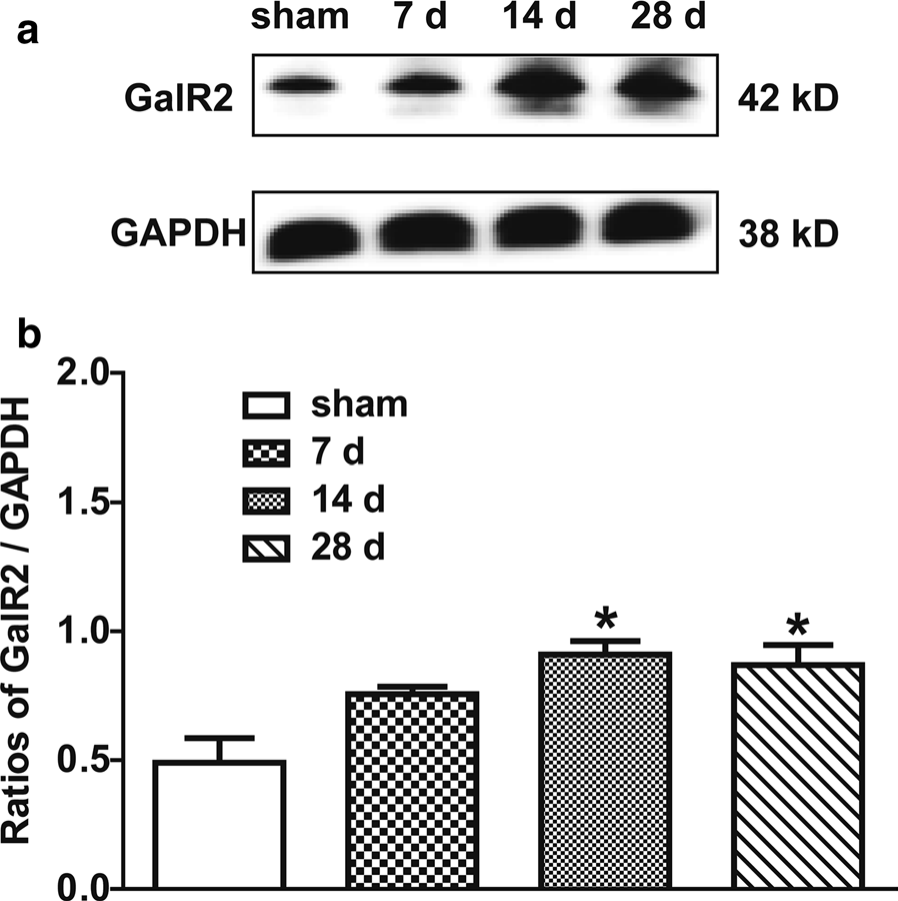
Expression of GalR2 in the NAc of CCI rats. **a** Representative Western blots for GalR2 and GAPDH in the sham-operated group and the CCI-induced neuropathic pain groups (7d: 7 days after CCI; 14d: 14 days after CCI; 28d: 28 days after CCI). **b** Histograms showing the ratios of GalR2/GAPDH. The data are presented as the mean ± SEM. *P < 0.05 compared with the sham control group

We first investigated whether the GalR2 was activated in NAc of rats with sciatic neuropathy. Bilateral NAc tissues were collected for Western blot assay, and it is found that compared to sham control group, GalR2 expressions in the NAc were up-regulated 14 days (*n* = 3; *t* = 4.370, *P* < 0.05) and 28 days (*n* = 3; *t* = 3.950, *P* < 0.05), but not 7 days (*n* = 3; *t* = 2.769, *P* > 0.05) after CCI, while there was no significant difference in the expression of GalR2 in NAc at 14 and 28 days after CCI (*t* = 0.420, *P* > 0.05), as shown in Fig. 1. The data were analysed using one-way ANOVA followed by Bonferroni's multiple comparison test. These results suggest that the expression of GalR2 in bilateral NAc is up-regulated from day 14 to day 28 after CCI.

### Effects of intra-NAc injection of GalR2 antagonist M871 on the galanin-induced increases in HWL and HWT of CCI rats

**Fig. 2:**
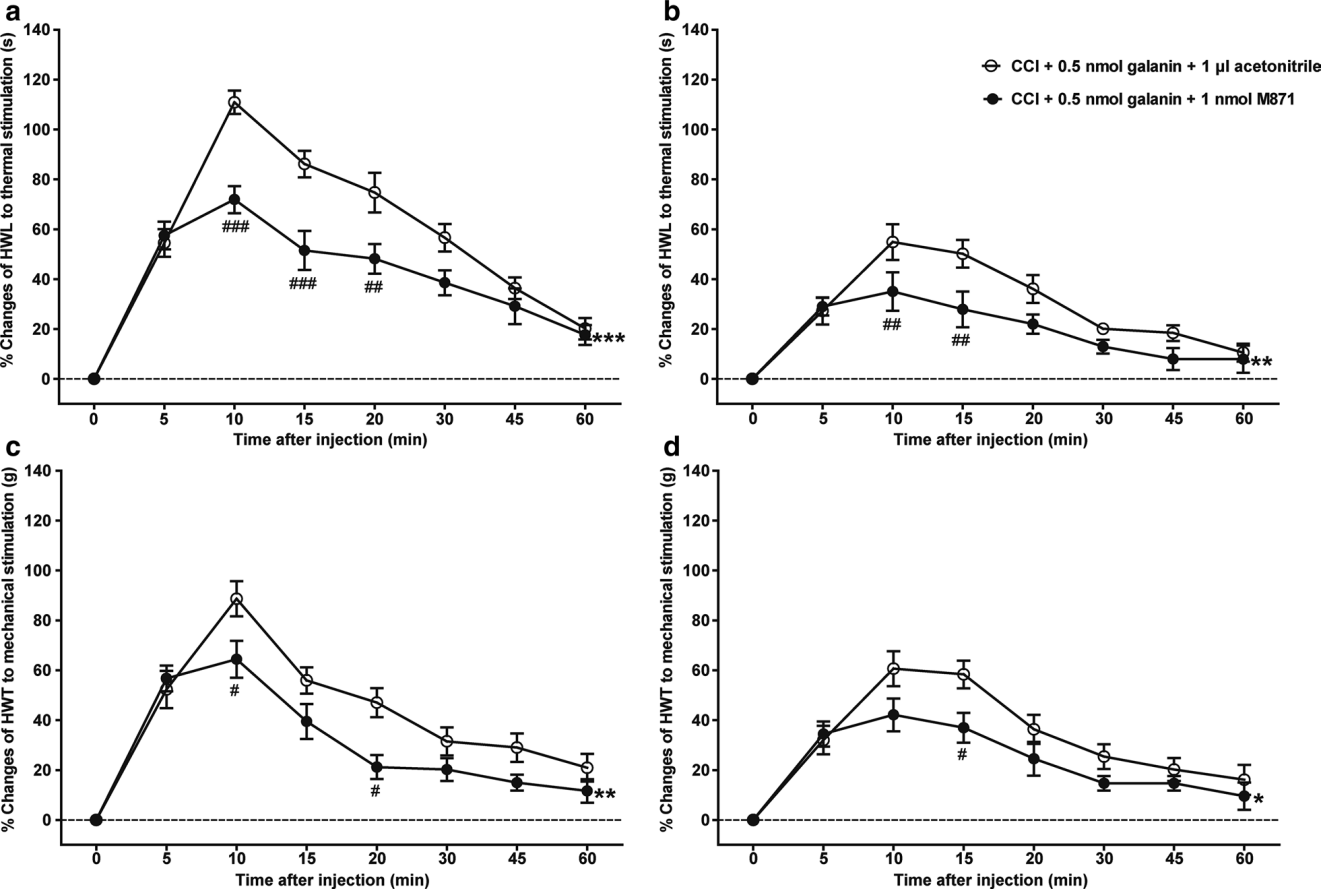
Effects of GalR2 antagonist M871 on the galanin-induced antinociception in NAc of CCI rats. **a** HWL of the left hind paw; **b** HWL of the right hind paw. **c** HWT of the left hind paw; **d** HWT of the right hind paw. 0.5 nmol galanin was bilaterally injected into NAc of CCI rats at 0 min, and 1 nmol M871 or 1 μl of acetonitrile as a control was bilaterally injected into NAc of CCI rats at 5 min. The data are presented as the mean ± SEM. *: Represents the overall difference between galanin + M871 group and galanin + acetonitrile group; #: Represents the difference between galanin + M871 group and galanin + acetonitrile group at each time point. ^#^, *P < 0.05, ^##^, **P < 0.01, ^###^, ***P < 0.001 compared with the acetonitrile group

Previous studies showed that galanin in the NAc had an antinociceptive effect via binding to GalRs in CCI rats [6]. To study whether the antinociceptive effect of galanin is mediated by the activation of GalR2, two groups of rats received an intra-NAc injection of 0.5 nmol galanin at 14 days after CCI, followed by an intra-NAc injection of 1 nmol GalR2 antagonist M871 (*n* = 8) or 1 μl of 3% acetonitrile (vehicle) as a control (*n* = 8) 5 min later. Compared with those of the galanin + acetonitrile group, the galanin-induced increases in HWL to thermal stimulation (left hind paw: *F*_(1,70)_ = 25.380, *P* = 0.0002; right hind paw: *F*_(1,70)_ = 9.218, *P* = 0.009) and HWT to mechanical stimulation (left hind paw: *F*_(1,70)_ = 12.370, *P* = 0.003; right hind paw: *F*_(1,70)_ = 6.020, *P* = 0.028) were reversed significantly by the intra-NAc injection of M871, as shown in Fig. 2. The data was analysed by two-way repeated-measures ANOVA followed by Bonferroni post-test. The results show that the antinociceptive effect of galanin in the NAc of CCI rats may be mediated by the activation of GalR2.

### Effects of intra-NAc injection of GalR2 agonist M1145 on HWL and HWT in CCI rats

**Fig. 3:**
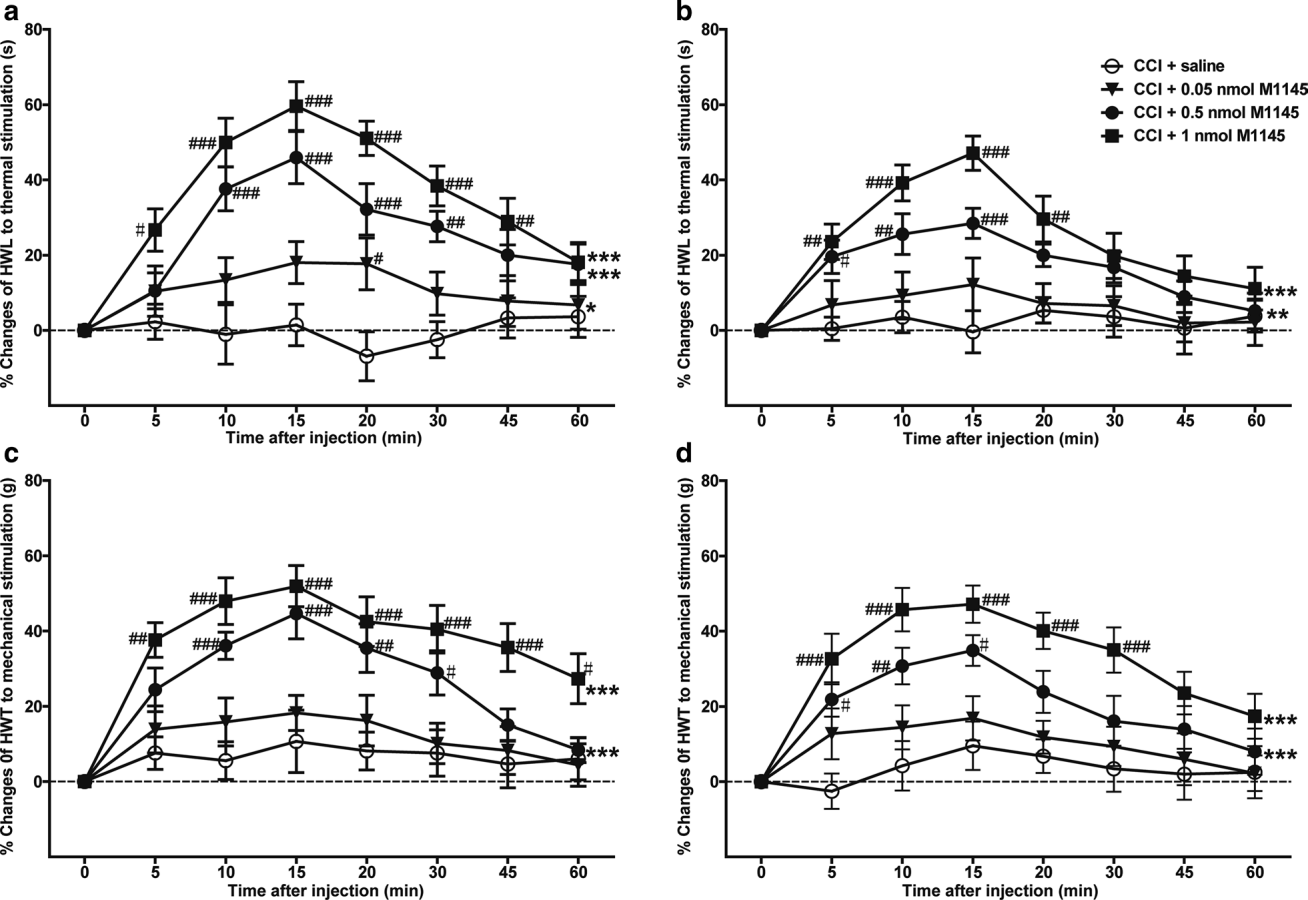
Effects of intra-NAc injection of M1145 on HWL and HWT of CCI rats. **a** HWL of the left hind paw; **b** HWL of the right hind paw. **c** HWT of the left hind paw; **d** HWT of the right hind paw. 0.05, 0.5, 1 nmol M1145 or 1 μl of saline as a control was bilaterally injected into NAc of CCI rats at 0 min. The data are presented as the mean ± SEM. *: Represents the overall difference between M1145 groups and saline group; #: Represents the difference between M145 groups and saline group at each time point. ^#^, *P < 0.05, ^##^, **P < 0.01, ^###^, ***P < 0.001 compared with the saline group

To study the antinociceptive effect of GalR2 activation on neuropathic pain, the rats received an intra-NAc injection of 1 μl of 0.05 nmol (*n* = 8), 0.5 nmol (*n* = 7), or 1 nmol (*n* = 8) GalR2 agonist M1145 or 0.9% saline as a control (*n* = 7) at 14 days after CCI. Compared with those of the CCI plus saline control group, the HWL to thermal stimulation and HWT to mechanical stimulation were significantly increased in the groups treated with 1 nmol (HWL: *F*_(1,78)_ = 58.290, *P*_left_ < 0.0001; *F*_(1,78)_ = 27.310, *P*_right_ = 0.0002. HWT: *F*_(1,78)_ = 83.710, *P*_left_ < 0.0001; *F*_(1,78)_ = 63.620, *P*_right_ < 0.0001) or 0.5 nmol (HWL: *F*_(1,72)_ = 33.360, *P*_left_ < 0.0001; *F*_(1,72)_ = 13.290, *P*_right_ = 0.003. HWT: *F*_(1,72)_ = 26.480, *P*_left_ = 0.0002; *F*_(1,72)_ = 22.590, *P*_right_ = 0.0005) M1145. In the group that received 0.05 nmol M1145, only the left HWL to thermal stimulation was significantly increased (HWL: *F*_(1,78)_ = 6.200, *P*_left_ = 0.027; *F*_(1,78)_ = 0.386, *P*_right_ = 0.545. HWT: *F*_(1,78)_ = 2.036, *P*_left_ = 0.177; *F*_(1,78)_ = 2.987, *P*_right_ = 0.108). The significance of the difference between the groups was determined by two-way repeated-measures ANOVA followed by Bonferroni post-test, as shown in Fig. 3. The results suggest that GalR2 agonist M1145 has an antinociceptive effect by activating GalR2 in the NAc of CCI rats.

### Comparison of the M1145-induced antinociceptive effect in NAc of rats before and after CCI

**Fig. 4:**
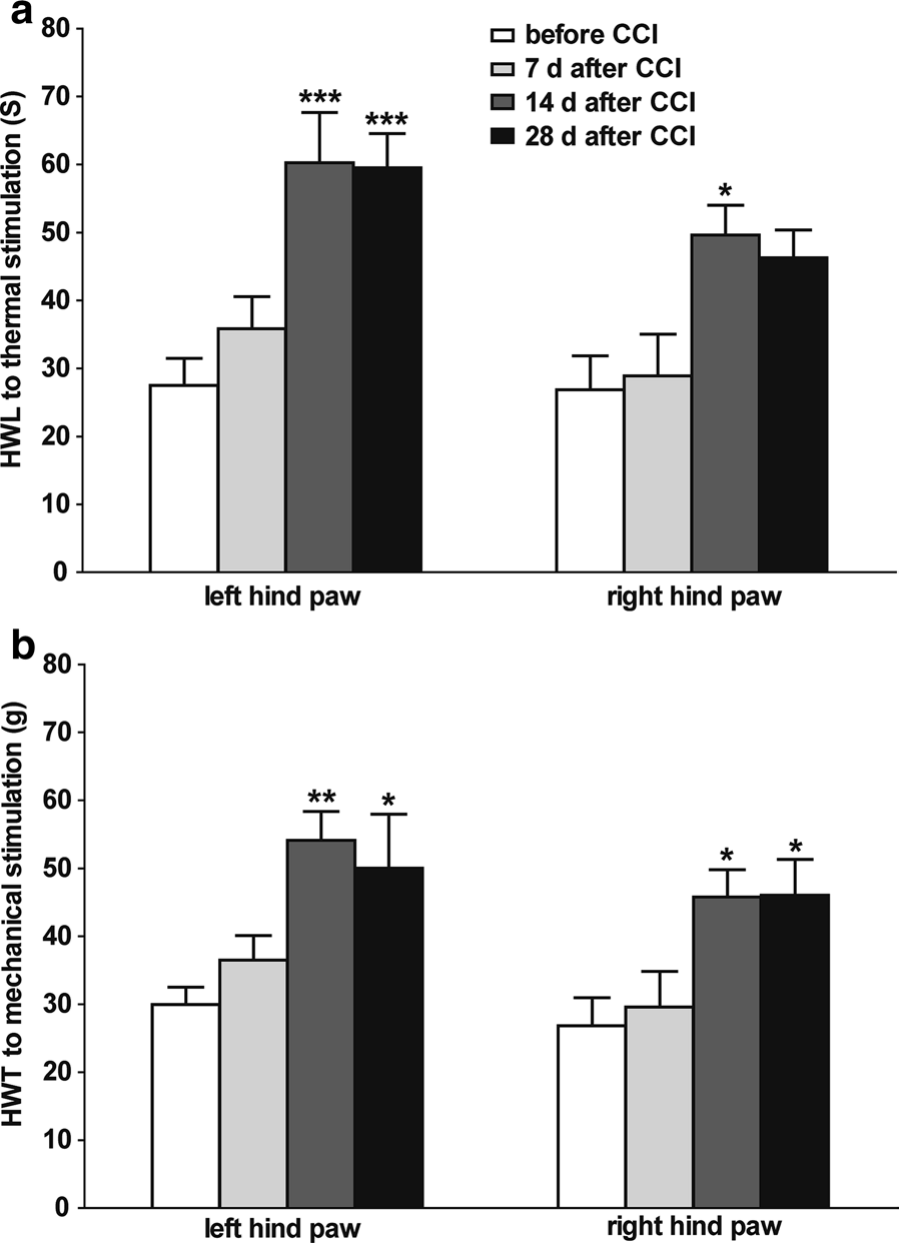
Comparison of the effects on HWL and HWT induced by M1145 in NAc of rats before and after CCI. The HWL and HWT of CCI rats were assessed 15 min after bilateral intra-NAc injection of M1145. The results are presented as the mean ± SEM. *P < 0.05, **P < 0.01 and ***P < 0.001 compared with that of before ligation

Because the previous results showed that the expressions of GalR2 in NAc were up-regulated from day 14 to day 28 after CCI (Fig. 1), therefore in order to test the accumulative effect of GalR2 agonist, four groups of rats, respectively, received intra-NAc injection of 1 nmol of GalR2 agonist M1145 before CCI (*n* = 10), or at day 7 (*n* = 7), day 14 (*n* = 7), day 28 (*n* = 7) after CCI. The HWL to thermal stimulation and HWT to mechanical stimulation were determined 15 min after the injection of M1145. Compared with the group of rats that received M1145 before CCI, both HWL and HWT increased significantly when M1145 were given at day 14 (HWL: *t*_left_ = 4.548, *P* < 0.001; *t*_right_ = 3.282, *P* < 0.05. HWT: *t*_left_ = 3.765, *P* < 0.01; *t*_right_ = 2.978, *P* < 0.05) and day 28 (HWL: *t*_left_ = 4.446, *P* < 0.001; *t*_right_ = 2.798, *P* > 0.05. HWT: *t*_left_ = 3.130, *P* < 0.05; *t*_right_ = 3.022, *P* < 0.05) after CCI, but there was no difference when M1145 was given at day 7 after CCI (HWL: *t*_left_ = 1.160, *P* > 0.05; *t*_right_ = 0.289, *P* > 0.05. HWT: *t*_left_ = 1.013, *P* > 0.05; *t*_right_ = 0.438, *P* > 0.05). Moreover, the increases in HWL and HWT induced by the intra-NAc injection of M1145 were not significantly different between day 14 and day 28 after CCI (HWL: *t*_left_ = 0.094, *P* > 0.05; *t*_right_ = 0.446, *P* > 0.05. HWT: *t*_left_ = 0.586, *P* > 0.05; *t*_right_ = 0.041, *P* > 0.05), as shown in Fig. 4. The differences between groups were determined by one-way ANOVA followed by Bonferroni's multiple comparison test. These results also imply that M1145 has an antinociceptive effect in the NAc of CCI rats, and this antinociceptive effect may be due to the activation of GalR2.

### Effects of intra-NAc injection of GalR2 antagonist M871 on M1145-induced increases in HWL and HWT of CCI rats

**Fig. 5:**
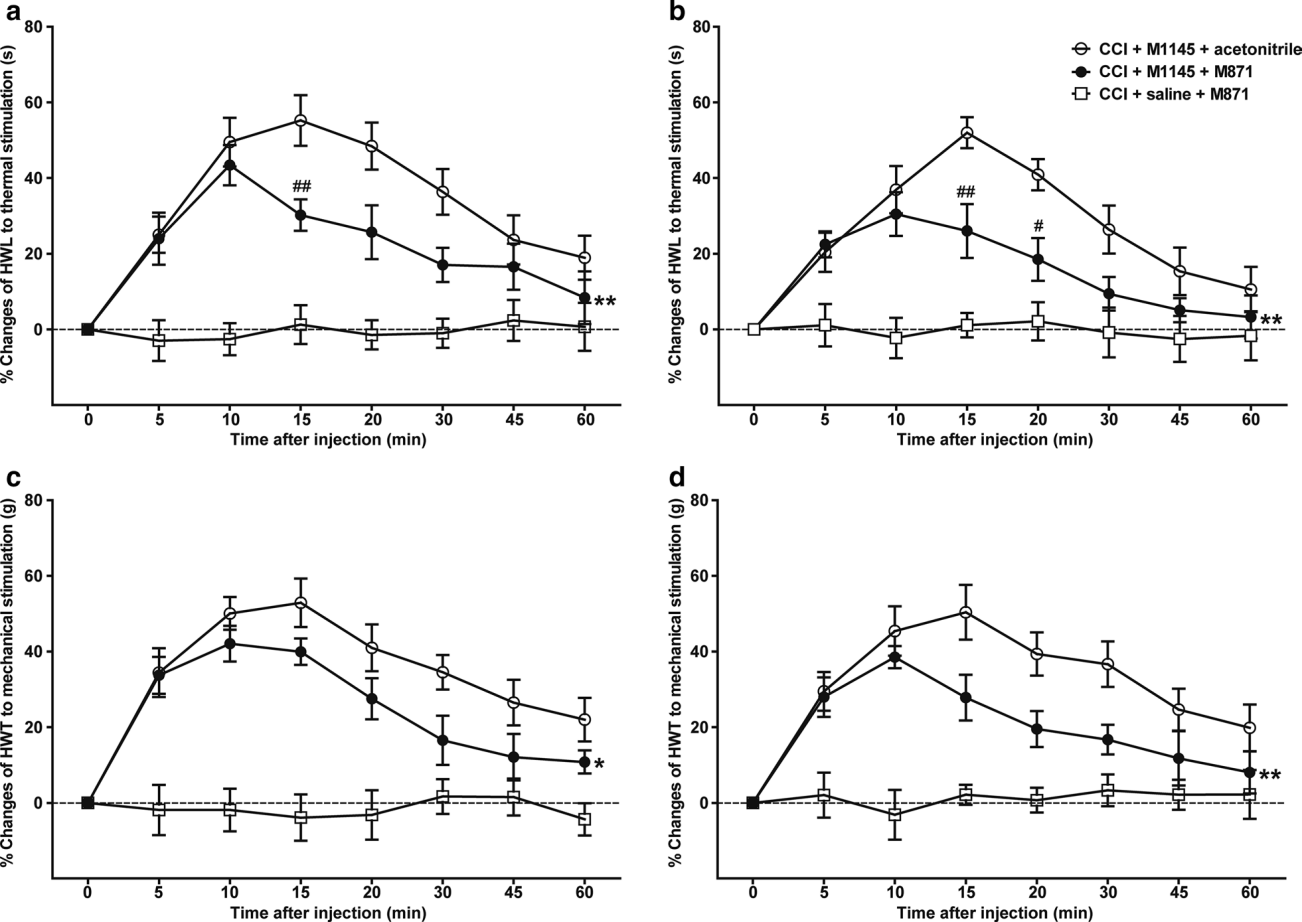
Effects of M871 on M1145-induced increases in the HWL and HWT in NAc of CCI rats. **a** HWL of the left hind paw; **b** HWL of the right hind paw. **c** HWT of the left hind paw; **d** HWT of the right hind paw. 1 nmol M1145 was bilaterally injected into NAc of CCI rats at 0 min, and 1 nmol M871 or 1 μl of acetonitrile as a control was bilaterally injected into NAc of CCI rats at 5 min. The data are presented as the mean ± SEM. *: Represents the overall difference between M1145 + M871 group and M1145 + acetonitrile group; #: Represents the difference between the M1145 + M871 group and the M1145 + acetonitrile group at each time point. ^#^, *P < 0.05, ^##^, **P < 0.01, ^###^, ***P < 0.001 compared with the acetonitrile group

To further study the role of GalR2 in neuropathic pain, two groups of rats received intra-NAc injection of 1 nmol GalR2 agonist M1145 14 days after CCI, followed by intra-NAc injection of 1 nmol of GalR2 antagonist M871 (*n* = 6), or 1 μl 3% acetonitrile as control (*n* = 7) 5 min later. As shown in Fig. 5, the M1145-induced increases in HWL to thermal stimulation (left hind paw: *F*_(1,55)_ = 16.890, *P* = 0.002; right hind paw: *F*_(1,55)_ = 14.160, *P* = 0.003) and HWT to mechanical stimulation (left hind paw: *F*_(1,55)_ = 8.820, *P* = 0.013; right hind paw: *F*_(1,55)_ = 12.480, *P* = 0.005) were significantly attenuated after the intra-NAc injection of M871, which suggested that the inhibition of GalR2 reversed the M1145-induced increases in HWL and HWT, and also indicated that M1145 might play an antinociceptive effect via binding to GalR2 in NAc of CCI rats. The significance of the difference between the groups was determined by two-way repeated-measures ANOVA followed by Bonferroni post-test.

In order to study the effect of intra-NAc injection of M871 alone, CCI rats were given an intra-NAc injection of 0.9% saline, followed 5 min later by intra-NAc injection of 1 μl of 1 nmol of M871 (*n* = 6), the HWL and HWT showed no marked changes during 60 min after the injection (Fig. 5).

### Comparison of the M1145-induced antinociceptive effect in NAc of intact rats and CCI rats

**Fig. 6:**
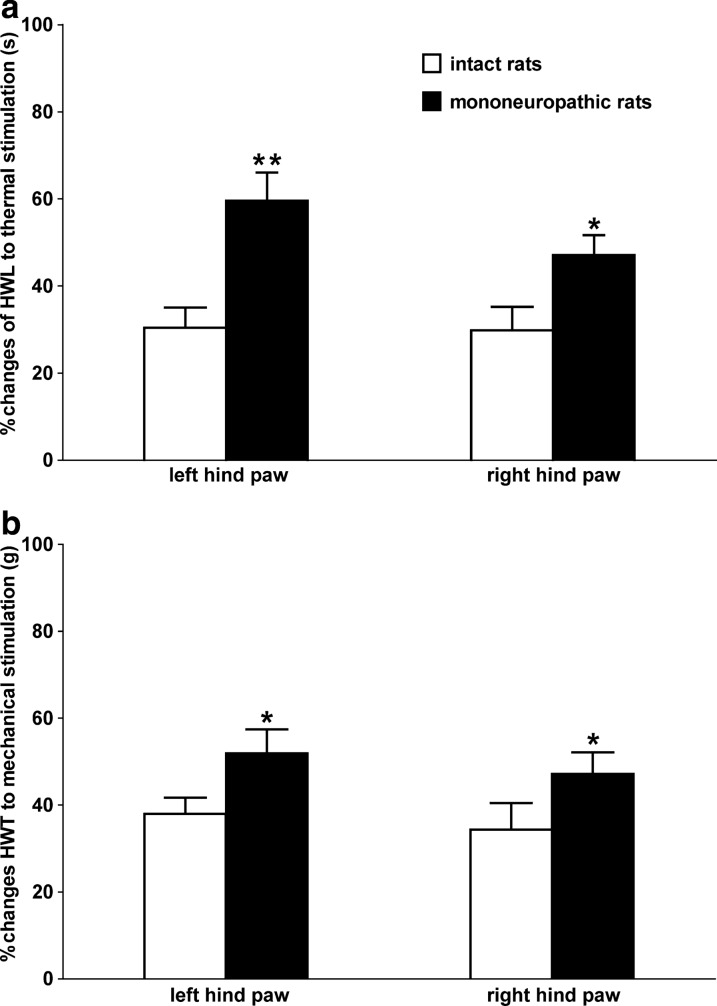
Comparison of the effects on HWL and HWT induced by M1145 in NAc of intact rats and CCI rats. The HWL and HWT of the CCI rats were assessed 15 min after the bilateral intra-NAc injection of M1145. The results are presented as the mean ± SEM. *P < 0.05 and **P < 0.01 compared with that of intact rats

On the day 14 after CCI, the rats received an intra-NAc injection of 1 nmol of GalR2 agonist M1145 (*n* = 8), and the intact rats received the same dose of M1145 (*n* = 10). The HWL and HWT peaked 15 min after the injection. Compared with that of the intact rats, as shown in Fig. 6, in CCI rats, the HWL to thermal stimulation and the HWT to mechanical stimulation increased significantly not only in left hind paw (HWL: *t* = 3.773, *P* = 0.017; HWT: *t* = 2.160, *P* = 0.046) but also in right hind paw (HWL: *t* = 2.370, *P* = 0.031; HWT: *t* = 2.138, *P* = 0.048). The differences between groups were determined by two-tailed Student's t-test. The results show that the antinociceptive effect of M1145 becomes more functional after CCI, this effect may be due to GalR2 activation in the NAc.

## Discussion

The results of this study showed that the expression of GalR2 was significantly up-regulated in bilateral NAc of rats after CCI (Fig. 1), and the intra-NAc injection of GalR2 antagonist M871 attenuated the galanin-induced increases in HWL to thermal stimulation and HWT to mechanical stimulation in CCI rats (Fig. 2), while the GalR2 agonist M1145 increased the HWL and HWT (Fig. 3). We also found that M1145-induced antinociceptive effect in NAc of rats 14 and 28 days after CCI was stronger than that of before CCI (Fig. 4), and M871 could block the M1145-induced increases in HWL and HWT of CCI rats (Fig. 5). Furthermore, M1145-induced antinociceptive effect in NAc of CCI rats was stronger than that in intact rats (Fig. 6), suggesting an involvement of GalR2 activation in the galanin-induced antinociceptive effects in NAc of CCI rats.

Galanin is expressed at the sites of pain mediation in the CNS, and both expression levels of galanin and its receptors were found to be increased in several models of neuropathy [8, 19, 20], implying that galanin is related to pain modulation in the CNS. A study illustrated that galanin analogue reduced pain behaviours in inflammatory, neuropathic, and acute pain models, and do not have dose-limiting toxic effects [21], thus it may be a potential drug for novel therapies.

Accumulated studies have shown that the NAc is one of the most important brain region involved in pain modulation. A study demonstrated that intra-NAc administration of calcitonin gene related peptide (CGRP) had an antinociceptive effect in rats and both mu- and kappa-opioid receptors were involved in the CGRP-induced antinociception [2]. A recent research reported that the infusion of N-acetylaspartylglutamate into the NAc significantly attenuated pain induced by the activation of sensory nerves through optical stimulation [5]. As early as 1992, Kordower et al. [22] reported that galanin-immunoreactive fibres were seen within the NAc of monkeys. Our recent study showed that galanin expression was up-regulated in the NAc after CCI [6]. Therefore, it is interesting to study whether galanin and its receptors play a role in pain modulation in the NAc of CCI rats.

Xu et al. (2012) demonstrated that GalR1 expression was up-regulated in spinal dorsal horn, whereas GalR2 was also up-regulated in both dorsal root ganglion and spinal dorsal horn after sciatic nerve-pinch injury [19]. Our previous study showed that the expression of GalR1 was up-regulated in the NAc of CCI rats, intra-NAc injection of GalR1 agonist M617 induced increases in HWL and HWT, implying that GalR1 mediates antinociceptive effect induced by galanin in NAc of CCI rats. However, the analgesic effect of M617 was weaker than that of galanin, suggesting that other GalRs may also be involved in pain modulating action of galanin in the NAc of CCI rats [12]. Zhang et al. reported that both the messenger ribonucleic acid (mRNA) level of GalR2 and the level of GalR2 in the ACC were increased in rats with acute inflammation [9], indicating a potential antinociceptive role of GalR2.

In this study, the result firstly showed that GalR2 expression in bilateral NAc was up-regulated 14 and 28 days after CCI (Fig. 1), then we employed GalR2 antagonist M871 to study the potential role of GalR2 in galanin-induced antinociceptive effect in the NAc of CCI rats, the result showed that intra-NAc injection of M871 attenuated galanin-induced increases in the HWL to thermal stimulation and the HWT to mechanical stimulation (Fig. 2). Consistent with this result, other studies found that M871 decreased galanin-induced antinociceptive effect in the periaqueductal grey (PAG) of rats [23] and in the ACC of normal rats or CCI rats [8] or rats with inflammatory pain [9].

The M1145 peptide shows more than 90-fold higher affinity for GalR2 over GalR1 and a 76-fold higher affinity over GalR3 [13]. To further investigate whether GalR2 in NAc is involved in the analgesic effect of galanin on neuralgia, we used M1145 to activate GalR2 and observe its effect on neuralgia. The results showed that intra-NAc injection of M1145 induced dose-dependent increases in the HWL to thermal stimulation and the HWT to mechanical stimulation in CCI rats (Fig. 3), and M871 reversed the M1145-induced increases in HWL and HWT (Fig. 5). Meanwhile the intra-NAc injection of M1145 increased both HWL and HWT significantly 14 and 28 days after CCI compared with that of the before CCI, but the increases in HWL and HWT were not significantly different at day 14 and day 28 after CCI (Fig. 4). These results imply that the GalR2 activation in the bilateral NAc from day 14 to day 28 after CCI may result from a strong antinociceptive effect of M1145. Moreover, our previous study demonstrated that the expression of galanin up-regulated in bilateral NAc of rats after CCI [6]. Therefore, in this study, M1145 was injected into bilateral NAc of intact rats or CCI rats. As a result, compared with that of the intact rats, the HWL and HWT increased significantly not only in left hind paw but also in right hind paw (Fig. 6), which further suggested that the antinociceptive effect of M1145 becomes more functional after CCI, this effect may be due to GalR2 activation in the NAc after CCI.

## Conclusion

This study demonstrated that the expression of GalR2 was up-regulated in bilateral NAc after CCI and the activation of GalR2 was involved in the galanin-induced antinociceptive effects in NAc of CCI rats.

## Data Availability

The datasets used and/or analysed during the current study are available from the corresponding author on reasonable request.

## Abbreviations

GalRs: Galanin receptors
NAc: Nucleus accumbens
HWL: Hind paw withdrawal latency
HWT: Hind paw withdrawal threshold
NMDA: *N*-Methyl-d-aspartic acid
CNS: Central nervous system
PAG: Periaqueductal grey
ACC: Anterior cingulate cortex
mRNA: Messenger ribonucleic acid
CGRP: Calcitonin gene related peptide
